# Do leaders walk the extra mile? The contribution of personal and work-related factors on daily step count increase in a university team step challenge

**DOI:** 10.3389/fpubh.2025.1648761

**Published:** 2025-09-29

**Authors:** Helena Manger, Katja Beck-Doßler, Olaf Hoos, Andrea Reusch, Andrea Szczesny

**Affiliations:** ^1^Department of Business Management, University of Würzburg, Würzburg, Germany; ^2^Conflict Management Service, University of Würzburg, Würzburg, Germany; ^3^Center for Sports and Physical Education, University of Würzburg, Würzburg, Germany; ^4^Healthy University Office, University of Würzburg, Würzburg, Germany

**Keywords:** health promotion, occupational health, exercise, walking, leadership, workplace intervention, team-based challenge

## Abstract

**Background:**

Sedentary work environments contribute to low physical activity (PA) levels, which are associated with adverse health and productivity outcomes. Workplace interventions such as step challenges offer a promising strategy to promote PA.

**Aim:**

This study investigates the effects of a six-week, team-based step contest conducted at a German university and examines personal and work-related factors including the role of leaders influencing PA.

**Methods:**

A one-group pre-post design was used to assess daily step counts of 331 participants across 44 self-formed teams during baseline, intervention and follow-up periods. Step data were collected via a mobile app, and a survey captured various demographic, work-related and intervention-related factors.

**Results:**

The step challenge significantly increased daily step counts by 1,700 on average compared to baseline. However, this increase was not sustained during follow-up. Males and older participants exhibited greater improvements. Notable, individuals in leadership positions showed a relatively greater increase in step counts during the intervention. However, the presence of a leader within a team did not significantly impact team colleagues’ performance. Other work-related factors such as work location and commute mode correlated with participants’ step counts but did not impact the step challenge’s effectiveness.

**Conclusion:**

Our findings suggest that team-based workplace interventions can effectively boost short-term PA but sustaining these improvements remains challenging and requires ongoing actions. Further, it is essential to take personal and work-related factors including the role of leaders into account to develop targeted strategies that enhance PA. Tailored strategies and organizational support are needed to promote long-term engagement. These insights may inform future workplace health initiatives aiming for sustainable impact.

## 1 Introduction

The decline in physical activity (PA) and the rise of sedentary work are strongly related to desk-based occupations as many employees spend most work hours sitting (1–4). Low activity levels contribute to decreased productivity, higher absenteeism, and increased healthcare costs (4, 5). Hence, the World Health Organization (WHO) recommends reducing sedentary behavior and increasing PA (6) to significantly counteract the risk for cardiovascular disease, diabetes, obesity, and mental health disorders (2, 7–11).

To address these challenges, workplace interventions have gained attention. Organizations increasingly adopt workplace health initiatives to promote PA and thereby aim to increase productivity, job satisfaction, and reduce absenteeism (12–16).

In this context step challenges, particularly those incorporating feedback and social comparison, have shown high potential (17–24). Team incentives may further enhance engagement (14, 25–29). Previous research on step challenges has shown that both financial incentives (26, 30) as well as non-monetary rewards increase PA in short-term but long-term sustainability is uncertain (31, 32). Outbalancing incentive-removal post-intervention on the one hand and increased awareness of PA benefits and habit formation on the other seem to be decisive for sustained improvements. Further, randomized controlled trials indicate that intervention success strongly depends on design and participant characteristics (33–35). Within the specific setting of higher education, a “healthy university concept” (36) includes PA promotion. Setting-specific team-based competitions with incentives have shown very promising increases of up to 4,800 steps per day (30). Besides, the ARK project in Norway demonstrated the effectiveness of holistic health promotion programs in higher education (36), and a systematic review of 17 studies found positive health outcomes in PA, weight management, and nutrition (37). However, diverse employee demographics and organizational structures present unique challenges for workplace interventions (38, 39) with time constraints, workplace cultures, and the complexity of integrating such programs into daily routines representing typical barriers (40). Besides, gender and age differences may influence changes in PA behavior. Men tend to be more motivated by competition, whereas women prioritize fitness and appearance (41, 42), and gender specific differences in motivation for PA diminish with age (43). Younger individuals are motivated by health and goalsetting (44). Also, factors like lifestyle, education and job characteristics influence activity levels (40, 45). Additionally, in the context of workplace PA interventions, leadership may influence behavior changes (16, 46, 47). Leaders shape organizational culture and often serve as role models (15, 48–50). Notably, participation in health interventions may also contribute to improvements of leadership (15, 51).

However, few studies have explored the role of leadership in the context of workplace PA interventions involving self-formed teams without designated intervention leaders. This gap is especially relevant in higher education institutions, where leadership structures differ significantly from those in the private sector. At universities, leadership is often distributed and exercised through indirect forms such as mentorship, coordination, and project management rather than through formal, hierarchical authority (52, 53). Understanding these unique leadership dynamics is critical for evaluating PA promotion interventions in academic environments. Additionally, recognizing personal and work-related factors seems crucial for designing effective workplace interventions for PA promotion. Therefore, this study examines the effectiveness of a six-week team step challenge using a gamified mobile app in increasing daily step counts among employees at a German university. Further this study explores individual, work-related, and team-related influencing factors. A particular focus is placed on the role of leadership, examining whether individuals in leadership roles show different outcomes, and whether their presence influences team performance.

We hypothesize that a non-monetary, team-based step contest will increase daily step counts compared to baseline levels, but we expect step counts to decline post-intervention. In addition, we examine how intervention effects relate to individual characteristics, and workplace dynamics. In addition, we expect individuals with leadership responsibilities to demonstrate greater engagement, and explore whether their presence affects overall team outcomes.

## 2 Methods

### 2.1 Study design and participants

This manuscript follows the Template for Intervention Description and Replication (TIDieR) checklist to ensure clear and replicable reporting of the intervention (54).

The intervention was a team-based step challenge, named “Team-Heroes,” in a German university. The goal of the intervention was to promote PA and foster team spirit among university employees.

Participants used a mobile app, which tracked steps via smart devices (55). Participant recruitment was done via university website and email. 331 individuals self-selected into 44 teams of 3–15 members. A survey with 15 items on participants’ demographics, lifestyle habits, job-related characteristics, and motives towards participation was conducted electronically as part of the registration along with participants consent for anonymous data collection and analysis.^1^ Of particular interest is the answer to the question of whether a person has personal responsibilities at work, indicating leadership roles. In addition, to better understand the leadership landscape, data from the university’s human resources (HR) system was collected. Step data were collected during a baseline period, a intervention period, and a follow-up period. The step-challenge app automatically synchronized step counts detected by the accelerometer of participants’ smart devices. During the intervention, participants received feedback on team step count and ranking as well as a map with a virtual journey via the app. No feedback was provided during baseline and follow-up phases. Members of the winner team received culture vouchers at the end of the intervention—gift certificates redeemable at local cultural institutions such as theaters or museums—and certificates of achievement, which were personalized documents listing the team’s name and final ranking, awarded to each team member. These prizes were not announced prior to the challenge.

The intervention was organized and coordinated by the university’s healthy workplace initiative team. The step challenge was delivered digitally through the mobile app and took place remotely. No specific physical infrastructure was required beyond access to a smartphone or wearable device. Data collection took place over a 12-week period from late August to mid-November 2023. The intervention lasted 6 weeks. The baseline and follow-up periods each lasted 3 weeks.

Teams were self-formed based on social or work relationships. The app features and challenge structure were standardized across all participants.

No modifications were made to the intervention during the study period. The intervention was delivered as planned.

All measures were taken to ensure participant confidentiality, anonymity, and privacy. The study was conducted in accordance with the declaration of Helsinki and was approved by the institutional review board (approval number: EV2025/1-0302).

### 2.2 Primary outcome and data treatment

We chose a one-group pre-post design. Participants’ daily steps served as primary outcome. Participants with no valid step data were excluded. To ensure data quality, extreme values (<1,000 or >35,000 steps/day) were treated as missing values (26, 45, 56), based on the distribution of our dataset, where the 5th percentile was 950 steps and the 99th percentile was 34,079 steps. Values outside this range were rare and potentially affected by device errors or manipulation. An individual-centered imputation method for missing step counts by using the average of individual’s weekday or weekend data (56) was applied separately to each phase (baseline, intervention, follow-up). For individuals who had no data available from any weekday or weekend within a specific period, we substituted the missing steps with the overall weekday or weekend average. With these procedures we obtained a balanced dataset of 27,804 observations from 331 participants in total. For variables related to participants’ self-reported characteristics, a worst-case scenario approach was applied (14). Every missing data point was treated as such and not imputed. Responses marked as “other” were treated as missing.

### 2.3 Statistical analysis

Besides descriptive statistics (mean ± standard deviations), one-way mixed analysis of variance (ANOVA) with repeated measures was conducted to assess changes in step counts across study periods (baseline, intervention, follow-up). *Post hoc* pairwise comparisons with Bonferroni correction were applied. Additionally, two-way ANOVA with repeated measures was used to examine differences in step counts and intervention effectiveness based on personal and work-related factors. Participants’ mean daily step counts served as dependent variable. If Levene’s test indicated a violation of the assumption of homogeneity of variances, the dependent variable was logarithmized. Pearson pairwise correlation analysis was performed to explore relationships between participants’ characteristics. Analysis was conducted using Stata Version 17.0.

## 3 Results

**Figure 1:**
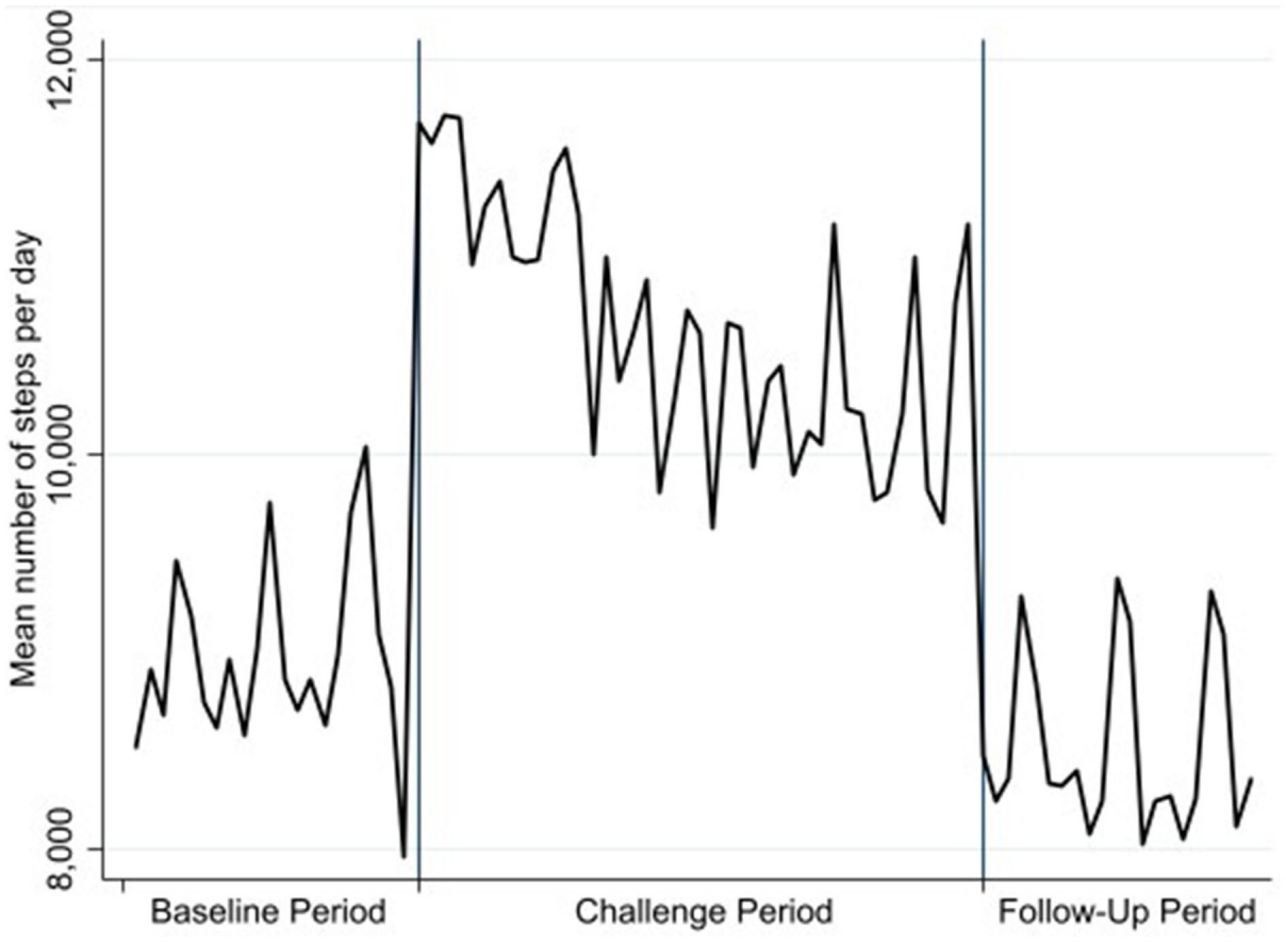
Average daily step counts.

Figure 1 visualizes dynamics of daily step counts throughout the study. Average daily step counts increased from 8,955 steps/day at baseline to 10,653 during the intervention, declining to 8,514 in follow-up (see Table 1). A one-way ANOVA using the logarithm of the step variable identified a significant main effect of the intervention [*F* (2, 660) = 42.15, *p* < 0.01, $\eta^{2}$ = 0.11]. *Post hoc* pairwise comparisons revealed that step counts were significantly higher during the intervention compared to baseline (*p* < 0.01) and follow-up (*p* < 0.01). The difference between baseline and follow-up was not statistically significant. A post-hoc one-way ANOVA showed a significant main effect of treatment weeks on step counts (*F* (5, 1,650) = 17.44, *p* < 0.01, $\eta^{2}$ = 0.05). *Post hoc* comparisons indicated a significant decline in step counts from the first to the later weeks of the intervention.

**Table 1:** Summary statistics.

Variable	Overall			Baseline period			Treatment period			Follow-up period		
	Obs.	Mean	Std. Dev.	Obs.	Mean	Std. Dev.	Obs.	Mean	Std. Dev.	Obs.	Mean	Std. Dev.
All study participants	27,804	9,694	5,902.24	6,951	8,955	5,506.48	13,902	10,653	6,398.61	6,951	8,514	4,834.05
Age												
16–24 years	2,772	9,381	5,519.42	693	9,435	5,739.47	1,386	9,822	5,832.80	693	8,443	4,436.21
25–34 years	13,272	9,911	5,931.46	3,318	9,231	5,620.09	6,636	10,881	6,414.07	3,318	8,650	4,790.29
35–44 years	5,460	9,436	5,899.30	1,365	8,837	5,496.22	2,730	10,293	6,377.04	1,365	8,320	4,963.64
45–54 years	3,276	9,512	5,806.96	819	8,148	4,954.88	1,638	10,553	6,332.13	819	8,795	5,066.36
55–65 years	2,268	9,557	6,145.91	567	8,126	5,173.23	1,134	11,272	6,688.40	567	7,558	4,799.04
Sex												
Female	16,380	9,466	5,738.38	4,095	8,967	5,417.72	8,190	10,257	6,225.11	4,095	8,381	4,711.20
Male	9,912	10,111	6,250.07	2,478	8,973	5,829.58	4,956	11,402	6,690.34	2,478	8,667	5,104.91
Living situation												
Alone	7,728	10,128	6,286.88	1,932	9,158	5,807.28	3,864	11,272	6,803.62	1,932	8,811	5,151.45
Shared apartment	3,612	9,464	5,483.76	903	9,186	5,322.20	1,806	10,369	6,047.78	903	7,933	3,847.70
With partner	672	8,566	4,737.32	168	8,393	4,208.60	336	8,811	5,131.81	168	8,250	4,402.25
With children & partner	6,720	9,827	6,180.15	1,680	9,325	6,005.21	3,360	10,631	6,621.89	1,680	8,722	5,127.35
With children	4,704	9,329	5,614.58	1,176	8,905	5,481.34	2,352	9,960	5,889.16	1,176	8,494	5,006.11
PA in free time												
No/little sport	6,804	8,436	5,350.41	1,701	7,244	4,387.57	3,402	9,614	6,056.20	1,701	7,273	4,047.40
Much sport	17,808	10,237	6,102.66	4,452	9,725	5,924.33	8,904	11,095	6,540.33	4,452	9,033	5,015.05
Personnel responsibility												
No	17,724	9,647	5,883.62	4,431	9,101	5,644.87	8,862	10,459	6,324.47	4,431	8,568	4,882.51
Yes	4,536	10,220	6,294.33	1,134	9,429	5,829.21	2,268	11,630	6,843.04	1,134	8,190	4,708.61
Individual with personnel responsibility in team												
No	6,972	8,995	5,905.26	1,743	8,600	5,792.47	3,486	9,678	6,411.71	1,743	8,025	4,660.60
Yes	20,832	9,928	5,882.87	5,208	9,074	5,402.75	10,416	10,980	6,361.21	5,208	8,677	4,880.26
Qualification												
Lower secondary	840	11,628	8,015.16	210	11,639	7,597.56	420	12,543	8,572.36	210	9,786	6,910.55
Upper secondary	3,864	8,696	5,116.06	966	8,179	4,916.94	1,932	9,425	5,649.78	966	7,752	3,808.46
Tertiary	15,876	10,067	5,982.35	3,969	9,368	5,743.59	7,938	11,010	6,392.15	3,969	8,882	4,979.08
Postgraduate	3,696	8,998	5,851.84	924	8,446	5,318.41	1,848	9,907	6,619.82	924	7,732	4,189.11
Commuting distance												
>3 km	18,396	9,489	5,906.72	4,599	8,645	5,408.46	9,198	10,442	6,421.51	4,599	8,426	4,918.02
<3 km	9,408	10,094	5,873.18	2,352	9,561	5,645.39	4,704	11,066	6,334.00	2,352	8,685	4,661.81
Means of transport for commuting												
Public transport	4,788	10,426	6,214.83	1,197	9,462	6,067.73	2,394	11,535	6,653.88	1,197	9,171	4,921.59
Bicycle	6,468	9,742	6,124.28	1,617	9,168	5,717.60	3,234	10,586	6,686.49	1,617	8,628	4,996.35
On foot	4,704	10,466	5,850.44	1,176	9,767	5,399.20	2,352	11,643	6,342.47	1,176	8,814	4,622.32
Car	8,652	8,862	5,497.36	2,163	8,319	5,251.95	4,326	9,656	5,923.04	2,163	7,816	4,528.98
PA at work												
Very little	3,696	9,711	6,178.70	924	9,033	6,258.71	1,848	10,514	6,593.54	924	8,783	4,903.92
Little	8,820	9,325	5,729.55	2,205	8,746	5,757.77	4,410	10,077	6,015.34	2,205	8,401	4,847.43
Sufficient	9,156	9,734	5,936.44	2,289	8,892	5,183.28	4,578	10,875	6,564.73	2,289	8,295	4,748.37
Much	2,940	10,365	5,967.78	735	10,110	5,807.93	1,470	11,229	6,435.33	735	8,894	4,717.97
Very much	336	13,935	6,813.16	84	12,349	4,997.57	168	16,035	7,735.54	84	11,322	4,857.61
Work area												
Science	13,356	9,879	5,969.10	3,339	9,429	5,677.09	6,678	10,686	6,447.40	3,339	8,716	4,930.87
Science support	10,584	9,799	6,102.09	2,646	8,717	5,640.95	5,292	10,963	6,580.79	2,646	8,552	5,009.66
Working from home												
0%	12,180	9,954	6,288.29	3,045	9,178	5,788.81	6,090	11,063	6,858.29	3,045	8,512	5,046.53
<20%	4,872	9,579	5,756.01	1,218	8,887	5,432.23	2,436	10,532	6,311.83	1,218	8,364	4,428.85
20–50%	5,796	9,554	5,551.04	1,449	8,827	5,584.98	2,898	10,344	5,817.41	1,449	8,701	4,680.93
>50%	1,428	8,842	5,395.14	357	8,558	5,261.22	714	9,202	5,689.51	357	8,406	4,861.20
Previous participation in step challenge												
No	19,740	9,233	5,764.72	4,935	8,718	5,518.66	9,870	10,056	6,228.19	4,935	8,103	4,691.37
Yes	6,300	11,057	6,190.48	1,575	9,733	5,796.22	3,150	12,474	6,579.76	1,575	9,547	5,009.92
Reason for signing up												
Team spirit	13,440	9,813	5,755.84	3,360	9,051	5,544.21	6,720	10,865	6,216.07	3,360	8,474	4,464.41
Persuaded by work colleagues	5,292	8,711	5,871.49	1,323	8,650	5,727.72	2,646	9,253	6,263.50	1,323	7,689	5,003.93
Exercise more	1,176	10,985	5,969.83	294	9,685	5,162.19	588	12,426	6,521.88	294	9,402	4,762.55
Fun, challenge	4,536	10,286	6,244.46	1,134	9,695	5,984.24	2,268	11,045	6,645.56	1,134	9,360	5,434.93
Team composition criterion												
Work relationships	19,236	9,957	6,067.56	4,809	9,215	5,723.77	9,618	10,939	6,557.97	4,809	8,734	4,952.17
Acquaintances, friendships	2,100	8,953	5,566.05	525	8,270	5,674.05	1,050	9,939	5,782.96	525	7,663	4,568.70
Habits of exercise, sportiness	336	12,247	7,744.47	84	12,704	7,585.89	168	12,955	8,438.06	84	10,372	6,040.19
Team size												
3–4 members	1,764	9,191	5,005.28	441	8,937	4,963.36	882	9,412	5,361.25	441	9,002	4,242.29
5–7 members	8,316	9,570	5,699.47	2,079	8,839	5,262.52	4,158	10,512	6,196.24	2,079	8,416	4,673.16
8–10 members	11,844	10,249	6,375.17	2,961	9,430	5,857.29	5,922	11,336	6,924.28	2,961	8,893	5,246.15
11–15 members	5,880	8,903	5,298.62	1,470	8,167	5,164.85	2,940	9,851	5,662.43	1,470	7,742	4,224.64

This table presents descriptive statistics for step counts overall and separately for the three periods: baseline, treatment, and follow-up. Mean and standard deviation for the imputed variable of individual daily step counts are displayed by various factors.

The mean age was 35.07 (±11.04) years. ANOVA results indicated no significant main effect of age on step counts (see Table 2), but older participants (55–65 years) exhibited the largest relative increase in daily step counts. Among participants, 62% were female (see Table 3). Baseline step counts were comparable between males and females, though males exhibited a greater increase during the intervention (see Tables 1, 2).

**Table 2:** Two-way ANOVA results.

Variable	Model	***ƞ* ^2^**	Factor	***ƞ* ^2^**	Period	***ƞ* ^2^**	Interaction	***ƞ* ^2^**	Levene’s test
Age	7.54(331)***	0.80	0.30(4)	0.00	53.03(2)***	0.14	2.13(8)**	0.03	0.24
Sex	7.59(316)***	0.79	1.20(1)	0.00	75.41(2)***	0.20	4.44(2)***	0.01	0.18
Living situation	7.80(288)***	0.80	0.43(4)	0.01	18.44(2)***	0.06	1.07(8)	0.02	0.17
PA in free time	6.47(296)***	0.77	17.97(1)***	0.06	35.39(2)***	0.11	1.85(2)	0.01	0.13 (log)
Personnel responsibility	8.45(268)***	0.81	0.41(1)	0.00	58.30(2)***	0.18	4.76(2)***	0.02	0.22
Individual with personnel responsibility in team	7.42(334)***	0.79	2.99(1)*	0.01	45.38(2)***	0.12	2.03(2)	0.01	0.12
Qualification	6.19(296)***	0.76	1.87(3)	0.02	10.01(2)***	0.03	0.42(6)	0.00	0.46 (log)
Commuting distance	6.11(334)***	0.76	2.84(1)*	0.01	38.88(2)***	0.11	1.06(2)	0.00	0.22 (log)
Means of transport	7.79(300)***	0.80	2.81(3)**	0.03	66.89(2)***	0.19	0.80(6)	0.01	0.50
PA at work	7.91(306)***	0.81	1.51(4)	0.02	25.54(2)***	0.08	1.15(8)	0.02	0.31
Work area	7.73(288)***	0.80	0.19(1)	0.00	63.18(2)***	0.18	2.89(2)*	0.01	0.30
Working from home	6.37(296)***	0.77	0.16(3)	0.00	15.34(2)***	0.05	0.90(6)	0.01	0.10 (log)
Previous participation in step challenge	7.83(313)***	0.80	11.11(1)***	0.03	69.07(2)***	0.18	5.22(2)***	0.02	0.38
Reason for signing up	8.21(298)***	0.81	2.05(3)	0.02	33.75(2)***	0.11	1.61(6)	0.02	0.37
Team composition criterion	8.00(263)***	0.80	1.56(2)	0.01	7.23(2)***	0.03	0.33(4)	0.00	0.14
Team size	6.04(338)***	0.76	1.48(3)	0.01	17.74(2)***	0.05	0.87(6)	0.01	0.23 (log)

This table presents the results of multiple two-way ANOVA tests examining differences in daily step counts across various demographic, work-related, and challenge-related factors. *F*-values are reported. Degrees of freedom are presented in brackets. Levene’s test for homogeneity of variances was conducted to test for equality of variance. If Levene’s test indicated a violation of the assumption of homogeneity of variances, the dependent variable was logarithmized. The “log” notation in the table indicates where this transformation was applied.

*Significance ≤0.1, **Significance ≤0.05, ***Significance ≤0.01.

**Table 3:** Participants’ characteristics.

Variable	Number	Proportion
Age	322	
16–24 years	33	10.25%
25–34 years	158	49.07%
35–44 years	65	20.19%
45–54 years	39	12.11%
55–65 years	27	8.39%
Sex	313	
Female	195	62.30%
Male	118	37.70%
Living situation	279	
Alone	92	32.97%
Shared apartment	43	15.41%
With partner	8	2.87%
With children & partner	80	28.67%
With children	56	20.07%
PA in free time	293	
No/little sport	81	27.65%
Much sport	212	72.35%
Personnel responsibility	265	
No	211	79.62%
Yes	54	20.38%
Individual with personnel responsibility in team	331	
No	83	25.08%
Yes	248	74.92%
Qualification	289	
Lower secondary	10	3.46%
Upper secondary	46	15.92%
Tertiary	189	65.40%
Postgraduate	44	15.22%
Short distance to university	331	
>3 km	219	66.16%
<3 km	112	33.84%
Means of transport for commuting	293	
Public transport	57	19.45%
Bicycle	77	26.28%
On foot	56	19.11%
Car	103	35.15%
PA at work	297	
Very little	44	14.81%
Little	105	35.35%
Sufficient	109	36.70%
Much	35	11.78%
Very much	4	1.35%
Work area	285	
Science	159	55.79%
Science support	126	44.21%
Working from home	289	
0%	145	50.17%
<20%	58	20.07%
20–50%	69	23.88%
>50%	17	5.88%
Previous participation in step challenge	310	
No	235	75.81%
Yes	75	24.19%
Reason for signing up	291	
Team spirit	160	54.98%
Persuaded by work colleagues	63	21.65%
Exercise more	14	4.81%
Fun, challenge	54	18.56%
Team composition criterion	258	
Work relationships	229	88.76%
Acquaintances, friendships	25	9.69%
Habits of exercise, sportiness	4	1.55%
Team size	331	
3–4 members	21	6.34%
5–7 members	99	29.91%
8–10 members	141	42.60%
11–15 members	70	21.15%

This table presents descriptive statistics for participants’ self-reported characteristics and team characteristics.

More than 72% of participants reported engaging in PA multiple times per week during their free time (see Table 3). Descriptive statistics indicated that active participants walked more across all periods (see Table 1). A significant main effect of PA in free time on step counts was observed, though no significant interaction effect with the intervention was found (see Table 2).

A key focus of this study was the role of leadership. Data from the university’s HR system indicated that in May 2025, 423 individuals in scientific positions held officially designated leadership roles, collectively responsible for 3,846 employees (excluding student assistants). In scientific support, 62 leadership positions were identified, overseeing 871 employees. Leadership roles cover a wide range of functions. In the scientific domain, these include professorial leadership, research group leaders, spokespersons of major research projects, deans, and central facility directors. In scientific-support, leadership roles encompass department and unit heads, project managers, heads of technical and administrative services, and coordinators of specialized services such as family support or equal opportunity offices. Among study participants, approximately 20% reported having personnel responsibilities at work (22.79% among scientific staff, 19.13% among science support staff) (see Table 3). While this factor did not exhibit a significant main effect on mean daily step counts, a significant interaction effect with the intervention was identified (see Table 2). Individuals in leadership roles demonstrated a significantly greater increase in daily step counts. 75% of participants were in teams with at least one member holding personnel responsibilities (see Table 3), yet the presence of such individuals within a team did not significantly influence the challenge’s effectiveness in increasing daily step counts (see Table 2). 37% of participants were in teams with one leader, 23% with two, 8% with three, and 7% with four leaders. The maximum proportion of team members with leadership roles was 50%.

Results suggested a trend, with participants living closer to the university walking more across all three periods (see Tables 1, 2). However, the ANOVA results revealed no significant interaction effect of commuting distance with the intervention (see Table 2). Similarly, means of transport for commuting had a significant main effect on step counts, but we found no significant interaction effect with the intervention (see Table 2). Bonferroni-adjusted pairwise comparisons revealed significantly lower step counts for car users compared to public transport users and those walking.

PA at work did not significantly affect steps, nor was there a significant interaction effect with the intervention (see Table 2). However, descriptive statistics suggested that individuals with higher PA tended to have higher step counts (see Table 1).

Participants were nearly evenly split between science staff and science support roles (see Table 3). While both groups had similar baseline step counts, science support staff showed a greater increase in daily steps during the intervention (see Tables 1, 2). In contrast, simple main effects analysis did not indicate a significant impact of work area on step counts.

First-time step challenge participants, who comprised the majority (76%) of the sample (see Table 3), walked significantly less in all periods compared to others, and the intervention was more effective among those with prior challenge experience (see Table 2). Additionally, prior participation was positively correlated with working in science support roles (*p* < 0.01) and negatively correlated with joining the challenge due to persuasion by colleagues (*p* < 0.01). There is a trend suggesting that those in leadership positions may be slightly more likely to have participated before (*p* = 0.09) (see Table 2).

The reason for signing up and team composition criteria did not significantly influence steps, nor did it impact intervention effectiveness (see Table 2). However, descriptive statistics indicated that those who reported to form teams due to exercise habits showed higher step counts across all periods (see Table 1).

Team size ranged from 3 to 15 members. ANOVA results did not show a significant relationship of team size to step counts (see Table 2). Descriptive statistics indicated that smaller teams showed the smallest increase in daily steps while teams with 8–10 members showed the largest increase in daily steps (see Table 1). However, all groups experienced a decline in steps during the follow-up period.

## 4 Discussion

Our study provides interesting insights into step count trends during a step challenge intervention. Participants walked more than double the average German population (57), reaching approximately 10,700 steps/day during the intervention. Even pre-intervention, participants were already quite active, averaging around 9,000 steps/day, consistent with research showing that workplace interventions often attract physically active individuals (34). Indeed, the majority of participants reported engaging in sports multiple times per week. While research suggests that less active individuals benefit more from PA promotion programs (34, 35), such interventions often fail to engage this group. Our findings highlight this participation bias and underscore the need for targeted strategies to attract less active employees.

The challenge led to an increase of about 1,700 steps/day, demonstrating its effectiveness in promoting activity. This rise aligns with previous workplace PA interventions (18, 21, 24) including team-based interventions (14, 19, 20, 25, 26, 29), though it is lower than the 4,799-step increase observed in a team competition with financial incentives (30). This smaller effect may be due to a higher baseline (8,955 vs. 5,959 steps/day), the absence of a daily step goal, or the lack of monetary rewards. Nonetheless, our findings confirm that team-based challenges can still drive meaningful behavior change. Notably, our step increase surpasses the modest effects reported in some studies (18–20).

Participants showed strong initial engagement, likely driven by novelty, followed by a gradual decline, suggesting potential fatigue or being incompatible with daily duties over longer terms. This pattern underscores the importance of monitoring and addressing factors that may contribute to decreasing activity over time. After the challenge, step counts dropped, indicating that while short-term interventions effectively boost activity, maintaining long-term habits may require additional strategies. Step counts were even lower than before the challenge, though not statistically significant, possibly due to recovery after an intense final push or a return to typical activity levels if participants had increased their steps during the baseline period in preparation for the challenge.

By utilizing the multi-campus higher education environment, marked by a diverse range of stakeholder roles and operational goals (38, 39), our study captures the influence of team-based competitions on PA within a heterogeneous organizational context. This context provides additional insights into variations in activity levels and the effectiveness of workplace interventions.

Sociodemographic differences were evident. Our results suggest that female work forces are more likely to participate in team-based step contests. However, males exhibited a greater step increase, likely due to their higher competitiveness (41–43, 58, 59). Age influenced the effectiveness of the intervention with older participants showing the highest relative step increase. This aligns with research showing that older adults are more driven by extrinsic motivation in PA (27, 41), while younger individuals may respond better to goal setting (44). Our study did not include a daily step goal, which has been a key motivator in other studies (34). Further, our findings do not indicate that family responsibilities hinder PA aligning with prior research (19, 60). In general, higher self-reported PA in free time positively correlated with daily step counts aligning with prior research (19, 45). However, while previous research suggests that less active individuals benefit more from such interventions (24, 34, 35, 61), our study does not support this for the university setting.

In addition, work-related characteristics played a substantial role. Our analysis confirms results of previous research showing a link between commuting and PA (42, 60), with car users walking less. Besides, working from home showed a trend of lower steps, possibly due to reduced incidental movement (3) but the results were not significant. As suggested by prior research, we found differences between academics and science-supportive employees (40). Participants in academic or scientific roles exhibited a lower step count increase compared to science support staff. Science support staff were also more likely to have participated in a previous step challenge, as indicated by the positive correlation with prior participation, suggesting a higher initial engagement with PA initiatives.

Additionally, leadership may play an important role in shaping behavior within workplace PA interventions (16, 46, 47). In the university context, personnel responsibility typically involves supervisory duties over staff or students. This includes tasks such as delegating work, monitoring performance, approving leave, and supporting professional development. Individuals with personnel responsibilities are often accountable for team outcomes and contribute to the functioning of academic or administrative structures. Leadership at universities is more complex than formal supervisory roles alone. It is shaped by relational dynamics, trust, and informal negotiations of influence and responsibility (52, 53). The diversity of leadership roles reflects the distributed and non-hierarchical nature of leadership in higher education. This complexity extends across both scientific and scientific-support domains, including positions such as deans, research group leaders, department heads, technical managers, and coordinators of institutional services. Such a nuanced leadership structure is particularly relevant when evaluating participation in university-wide initiatives like team-based health promotion programs. In these contexts, the presence and engagement of individuals in leadership roles may influence participation and outcomes through role modeling, team motivation, or cultural signaling (15, 16, 46, 48–50).

Data from the university’s HR system shows that approximately 9% of employees hold formal leadership roles—9.91% in scientific positions and 6.65% in scientific-support roles. In contrast, 20% of study participants reported having personnel responsibilities, suggesting that individuals in leadership roles may be more inclined to engage in such health initiatives. Among scientific staff in the study, the figure was even higher (22.79%), aligning with institutional trends. This overrepresentation suggests that workplace PA interventions may appeal especially to individuals with leadership responsibilities.

It is also possible that the discrepancy between reported and formal leadership roles reflects the unique leadership culture in universities. Given the distributed and often informal nature of academic leadership (52, 53), individuals may perceive themselves as having personnel responsibility even in the absence of formal supervisory roles. Such perceptions can still influence engagement in workplace health initiatives, as informal leadership may foster a sense of responsibility, role modeling, or team cohesion.

Taken together, these findings suggest that workplace PA interventions may particularly appeal to individuals who see themselves in leadership roles—whether formally recognized or not. Survey results support this trend, showing that participants in leadership roles were more likely to have taken part in previous step challenges.

During the intervention, these individuals also showed greater increases in step count—possibly reflecting a desire to serve as role models or to support team cohesion (48, 49). Leaders may also be more accustomed to setting and pursuing goals, making them particularly responsive to structured, gamified health programs (62, 63). Another possible explanation is that individuals in leadership roles may have experienced increased pressure to perform due to their visibility within the team. This perceived accountability could have driven them to increase their activity levels, independent of intrinsic motivation or role modeling intentions.

Despite these findings, our results indicate that the mere presence of a leader in a team did not significantly affect other team members’ outcomes. This could be because the step challenge primarily relied on individual motivation rather than leadership-driven encouragement (46). Leaders may have focused on their own performance rather than actively fostering participation within their teams. Lack of leadership support may hinder PA promotion (16, 46, 47). Research highlights that there is a need to equip leaders with knowledge to foster participation and engagement (64). To leverage leadership more effectively in workplace PA interventions, organizations should implement strategies that actively involve leaders in promoting engagement within their teams. These insights highlight the importance of understanding how leaders’ PA behaviors may influence the effectiveness of workplace PA promotion interventions as pointed out in prior research (15, 38, 47).

Although some teams included multiple individuals with leadership responsibilities, there were no teams composed exclusively of leaders. The maximum proportion of leadership roles within a team was 50%, suggesting that leaders did not form separate, leader-only teams but were instead integrated into mixed-role groups. It is also plausible that some teams included multiple leadership levels (e.g., institute heads, professors, and research group leaders).

Further, participants with prior experience in step challenges had higher baseline step counts and demonstrated a greater increase in activity levels, highlighting a potential predisposition toward competition or intervention-driven PA – an observation that aligns with previous research (25).

Team composition might also play a role. Participants that joined teams based on shared exercise habits showed on average higher step counts across all three study periods. Team size ranged from 3 to 15 members, similar to earlier studies (19, 26). Teams with moderate size exhibited higher step counts.

Despite these insights, limitations must be acknowledged. Self-selection may have introduced bias and the reliance on participants’ step-tracking devices and self-reported data could affect the accuracy of the obtained data. Additionally, other forms of PA (e.g., cycling, swimming, yoga, aerobics) were not captured, as the app only recorded walking—i.e., step-based movement detected by smart devices. The predominantly female, office-based sample and short follow-up period also limit generalizability and long-term conclusions. Furthermore, it is unclear whether the teams in the study were strictly work teams, and whether the individuals reporting to have personnel responsibilities were formal, work-related leaders of their team colleagues. Finally, while we excluded extreme step values (<1,000 or >35,000 steps/day) to reduce potential measurement error, this decision may have led to the omission of valid but rare activity patterns. However, a robustness check including these values yielded comparable results, suggesting limited impact on the overall findings. A key limitation of this study is the absence of a control group, which restricts our ability to attribute the observed increase in step count solely to the intervention. The challenge took place in autumn 2023, with the baseline phase occurring during the semester break and the follow-up during the academic semester. External factors such as seasonal weather changes, academic workload fluctuations, and working-from-home patterns may have influenced participants’ activity levels regardless of the intervention. Future studies should consider including a control group to better isolate the effects of the intervention.

## 5 Conclusion

This study highlights the potential of a six-week team-based step challenge on university employees’ PA with an average increase of almost 20% in daily step count. However, the effectively boosted short-term activity was not maintained post-challenge. Further, step count increase was modulated by personal, work-related and team composition factors. Most beneficial outcomes might be achieved for male and older individuals, for leaders and science support staff, for those with prior experience and already active subjects, while several other groups in the higher educational setting may not be attracted in a similar way.

This strengthens the notion that both diverse employee demographics and organizational structures as well as the complexity of integrating PA enhancing interventions into daily routines represent typical barriers for the higher educational setting (38, 40).

A particularly notable insight from our study is the role of leadership on PA changes. Leaders demonstrated higher increases in step counts, likely due to strong goal orientation or a desire to lead by example. However, their presence alone did not significantly trigger team-wide activity levels, suggesting that leadership alone is not sufficient to drive collective behavior change. Actively involving and equipping leaders to support and motivate their teams might enhance the effectiveness of future programs.

Future research should therefore explore how different leadership styles influence participation and engagement in workplace PA programs. Interventions that actively involve and equip leaders to support and motivate their teams may enhance the overall impact of such programs. Additionally, strategies are needed to reach the less active individuals and more sedentary groups, as well as ways to keep them engaged and motivated throughout the challenge. A more diverse participant pool would additionally enlighten our understanding of how different demographic groups engage in PA challenges. Additionally, given the rising prevalence of remote work, developing tailored interventions for this population is increasingly important. Research is also needed to identify ways to sustain PA gains.

For institutions, these results suggest that short-term, low-cost digital interventions can be effective in initiating PA improvements. However, for long-term impact, such programs should be embedded into broader workplace health promotion strategies. This includes leadership training to support team motivation, personalized approaches for different employee groups, and support for integrating PA into daily routines.

Overall, our findings highlight the complexity of influencing factors on PA behavior and emphasize the need for personalized, sustainable interventions to promote long-term activity across diverse populations and how this is related to leadership support.

## Data Availability

The raw data supporting the conclusions of this article will be made available by the authors, without undue reservation.

## Footnotes

^1^Examples of survey questions (originally in German) include: “Do you have personnel responsibility at work?” (Yes/No), “How do you usually commute from home to workplace?” (On foot/By bike/By public transport/By car), and “On how many days per week do you regularly engage in physical activity in your free time for at least 30 min?” (No regular activity/1 day/2–3 days/4–5 days/6–7 days).
